# Structure–Activity Relationship of *Plesiomonas shigelloides* Lipid A to the Production of TNF-α, IL-1β, and IL-6 by Human and Murine Macrophages

**DOI:** 10.3389/fimmu.2017.01741

**Published:** 2017-12-11

**Authors:** Marta Kaszowska, Marta Wojcik, Jakub Siednienko, Czeslaw Lugowski, Jolanta Lukasiewicz

**Affiliations:** ^1^Hirszfeld Institute of Immunology and Experimental Therapy, Polish Academy of Sciences, Wroclaw, Poland; ^2^Department of Biotechnology and Molecular Biology, University of Opole, Opole, Poland

**Keywords:** *Plesiomonas*, lipid A, lipopolysaccharide, proinflammatory cytokines, THP-1, BMDM

## Abstract

*Plesiomonas shigelloides* is a Gram-negative bacterium that is associated with diarrheal disease in humans. Lipopolysaccharide (LPS) is the main surface antigen and virulence factor of this bacterium. The lipid A (LA) moiety of LPS is the main region recognized by target cells of immune system. Here, we evaluated the biological activities of *P. shigelloides* LA for their abilities to induce the productions of proinflammatory cytokines (TNF-α, IL-1β, and IL-6) by human and murine macrophages [THP-1 macrophages and immortalized murine bone marrow-derived macrophages (iBMDM)]. Four native *P. shigelloides* LA preparations differing in their phosphoethanolamine (PEtn) substitution, length, number, and saturation of fatty acids were compared with *Escherichia coli* O55 LA. The bisphosphorylated, hexaacylated, and asymmetric forms of the *P. shigelloides* and *E. coli* LA molecules had similar activities in human and murine macrophages, indicating that shortening of the acyl chains in *P. shigelloides* LA had no effect on its *in vitro* activities. The PEtn decoration also had no impact on the interaction with the toll-like receptor 4/MD-2 receptor complex. The heptaacylated form of *P. shigelloides* LA decorated with 16:0 exhibited strong effect on proinflammatory activity, significantly decreasing the levels of all tested cytokines in both murine and human macrophages. Our results revealed that despite the presence of shorter acyl chains and an unsaturated acyl residue (16:1), the bisphosphorylated, hexaacylated, and asymmetric forms of *P. shigelloides* LA represent highly immunostimulatory structures.

## Introduction

Lipopolysaccharide (LPS, an endotoxin), which is the main virulence factor of Gram-negative bacteria, including *Plesiomonas shigelloides*, is a well-characterized pathogen-associated molecular pattern and a powerful activator of the innate immune response. The LPS molecules of smooth bacteria are composed of three distinct regions: hydrophobic lipid A (LA), a core oligosaccharide, and the O-specific polysaccharide. The LA region critically affects the biological activity of endotoxins by mediating the interaction of LPS with pattern recognition receptors, such as toll-like receptor 4 (TLR4) (1), on monocytes/macrophages. As a result, signaling pathways are triggered followed by transcription factors activation (e.g., NF-κB) and production of proinflammatory cytokines (e.g., TNF-α, IL-1β, IL-6) that initiate and shape the host’s immune response against the pathogen. In the case of bacteremia caused by Gram-negative bacteria, an excessive response to LPS may lead to sepsis and septic shock (2–4).

**Figure 1:**
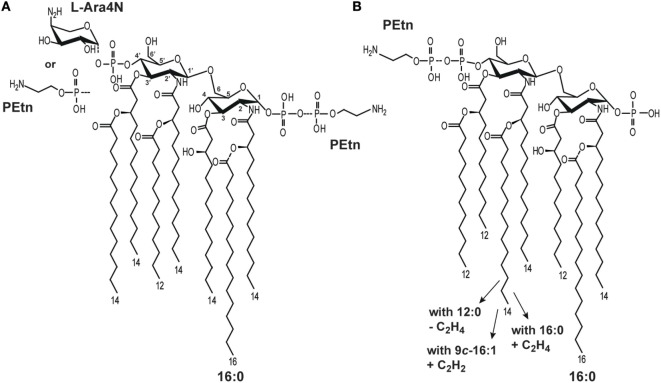
Structural heterogeneity of **(A)** *Escherichia coli* (5) and **(B)** *Plesiomonas shigelloides* lipid A (LA) molecules (8–10).

Bacteria produce LA molecules that are generally conserved, but show some structural variability in terms of the following: the number, length, and saturation of fatty acids; asymmetry; the carbohydrate backbone composition; and the presence of additional substituents, such as phosphate groups (P), ethanolamine (Etn), phosphoethanolamine (PEtn), 4-amino-4-deoxy-l-arabinose (l-Ara4N), and d-galacturonic acid (d-Gal*p*A) (1, 2). Structurally different LA molecules show different potencies in activating the mammalian macrophages (5). *E. coli* LA, which displays strong immunostimulatory or endotoxic activity in the mammalian host (6), consists of a bisphosphorylated carbohydrate backbone substituted with six asymmetrically distributed fatty acids (Figure 1A) (1, 7).

Several important pathogenic bacteria (i.e., *E. coli, K. pneumoniae, Shigella flexneri, Yersinia pestis, Neisseria meningitidis*) have been shown to modify their LA in ways that significantly alter TLR4-dependent signaling (1, 11). Such modifications include the expression of enzymes responsible for palmitoylation (leading to heptaacylated LA) (12) and the additions of P, PEtn, and/or l-Ara4N (Figure 1A) (1). Thus, it has been hypothesized that the expression of different LA types may be a mechanism through which pathogens modulate or evade the host immune response (6).

The bacterium, *P. shigelloides*, is associated with diarrheal disease in humans. It has been implicated in gastroenteritis outbreaks among tropical travelers and in patients who have ingested contaminated food or water, and reportedly stands as the third most common cause of diarrhea among travelers in Japan and China (13). *P. shigelloides* causes acute bacterial gastroenteritis and extra-intestinal infections, such as sepsis, meningitis, cellulitis, and septic arthritis (14). The extra-intestinal diseases caused by *P. shigelloides* are associated with a high mortality rate, even when patients are given appropriate antibiotic therapy and intensive care (15, 16).

The structures of *P. shigelloides* LPS, including that of the LA region, have been extensively studied. Natural *P. shigelloides* LA molecules comprise a mixture of structures that differ in the numbers, lengths, and saturations of their acyl chains, as well as in PEtn substitutions. Three strains (O-serotypes) have been characterized to date with reference to LA among 85 O-serotypes of *P. shigelloides* (17–19), and detailed structural analyses suggest that there is significant conservation of this region among all studied O-serotypes. In general, the LA of *P. shigelloides* comprises a β-Glc*p*N4P-(1 → 6)-α-Glc*p*N1P disaccharide backbone substituted with the following primary fatty acids: 14:0[3-(*R*)-OH] at N-2 and N-2’ and (*R*)-3-hydroxydodecanoic acid [12:0(3-OH)] at O-3 at O-3’. The heterogeneity of *P. shigelloides* LA reflects that the acyl residues at N-2’ and O-3’ are substituted with the following secondary acyls: (1) *cis*-9-hexadecenoic acid (9*c*-16:1) or hexadodecanoic acid (16:0) at N-2’ and 12:0 at O-3’ (strain dependent); (2) 14:0 at N-2’ and 12:0 at O-3’; and (3) 12:0 at N-2’ and O-3’. Other types have additional substitutions by PEtn and 16:0 as a seventh acyl chain (17–19) (Figure 1B). The LA of *P. shigelloides* differs from those of *E. coli* mainly in the length and saturation of their fatty acids.

Our previously published report discussed the biological *in vitro* and *in vivo* activity of *P. shigelloides* (serotype O54) LPS, which was found to have a stronger effect on cytokine production by murine macrophages and 10-fold higher *in vivo* toxicity in the actinomycin D-sensitized mouse model, compared to *E. coli* O55 LPS (19). We concluded that the non-typical structures of the core oligosaccharide, which lacks phosphate residues, and the LA, which has shorter fatty acid residues than *E. coli* LA, might be responsible for the higher biological activity. This work provided some interesting insight into the structure–*in vitro* activity relationships of *P. shigelloides* LA in comparison with *E. coli* LA.

To avoid the potential for the heterogeneity of LA molecules to influence their biological activity in an experimental setting, structure–activity relationships LA-based studies are usually performed using chemically synthesized LA analogs, such as tetra-, penta-, hexa-, and heptaacylated *E. coli* LA (8, 9, 20–24), or LA isolated from genetic mutants (10, 25, 26). Since synthetic LA analogs and mutated strains are not commercially available for *P. shigelloides*, we screened 85 *P. shigelloides* O-serotypes (unpublished results) and identified four strains that showed relatively low LA heterogeneity, and whose typical LA structures showed between-strain differences with respect to their palmitoylation and PEtn substitution. In the present study, we used the well-defined LA molecules from these four *P. shigelloides* strains to examine the effect of structural heterogeneity on the biological response triggered by LA in human (THP-1) and immortalized murine (iBMDM) macrophages. Human and murine models have been chosen, since species-specific recognition was demonstrated for TLR4/MD-2 complexes (4, 27).

This is the first report to assess the structure–*in vitro* activity relationships of *P. shigelloides* LA with respect to inducing the production of proinflammatory cytokines (TNF-α, IL-1β, and IL-6). We demonstrate how variations in the structure of *P. shigelloides* LA (the length of the acyl groups, the presence of PEtn, and the seventh acyl chain) affect its recognition by human and murine TLR4/MD-2 receptor complexes.

## Materials and Methods

### Bacterial Strains and Growth Conditions

Bacteria of *P. shigelloides* serotypes O14, O30, O61, and O75 and *E. coli* O55:B5 [Polish Collection of Microorganism (PCM)-224] were obtained from the PCM of the Hirszfeld Institute of Immunology and Experimental Therapy PAS (Wroclaw, Poland). Lyophilized bacteria were reconstituted, and strains were cultured on agar plates. Bacteria were washed from the solid medium with buffered phosphate saline (PBS) and used to inoculate liquid medium (Davis, 1.5 l). After 48 h of culture at 37°C, the bacteria were killed with 0.5% phenol, centrifuged (4000 × *g*/30 min/4°C), resuspended in water, and lyophilized.

### Cell Lines and Reagents

The human monocytic cell line THP-1 was purchased from the European Collection of Authenticated Cell Cultures (ECACC). immortalized murine bone marrow-derived macrophages (iBMDM) from wild-type mice (iBMDM WT), and TLR4 knockout mice (iBMDM TLR4^−/−^) were obtained from BEI Resources.

### Microextraction and Analysis of LA

The rapid, small-scale procedure used to directly isolate the LA molecules from freeze-dried *P. shigelloides* and *E. coli* O55 cells was performed as described previously with slight modifications (28). Briefly, bacterial cells (10 mg) were suspended in 400 µl of 1 M isobutyric acid-ammonium hydroxide (5:3, v/v), vortexed (20 min), sonicated (20 min), and incubated for 2 h at 100°C. The mixture was cooled in ice water and centrifuged (2000 × *g*/15 min/4°C). The supernatant was diluted with water (300 µl) and lyophilized. The samples were washed twice with 400 µl of methanol and centrifuged (2000 × *g*/15 min/4°C). Finally, the LA-containing sediment was suspended in water and lyophilized. To obtain *E. coli* LA, the LPS of *E. coli* O55 (Sigma-Aldrich) was subjected to mild hydrolysis with 1.5% acetic acid (15 min/100°C) as described previously (29). The LA-containing sediment was washed, resuspended in water, and freeze-dried. All LA samples were analyzed by matrix-assisted laser-desorption/ionization time-of-flight (MALDI-TOF) mass spectrometry (MS) using an UltrafleXtreme (Bruker, Germany) instrument. The MALDI-TOF MS spectra of LA were obtained in the negative ion mode. 2′,4′,6′-Trihydroxyacetophenone [10 mg/ml in 1:1 AcN/mQ (v/v)] was used as a matrix. Each LA sample was tested for the presence of potential immunostimulatory impurities, such as other lipids, proteins, peptides, and nucleic acids, using MALDI-TOF MS and the appropriate matrix (2′,4′,6′-trihydroxyacetophenone for lipids and nucleic acids, sinapinic acid for proteins, and α-cyano-4-hydroxy cinnamic acid for peptides).

### LA Solubilization for *In Vitro* Assays

Lipid A was dissolved in water (1 mg/ml) by the addition of triethalamine (TEA) (5 µl/ml) and vortexed to obtain a stock solution (1 mg/ml, pH 8.1). For stimulation experiments, the LA stock solution was diluted with culture medium. Prior to cell stimulations, all LA samples were analyzed by MALDI-TOF MS within the range of *m/z* 500–3000 to validate the LA structures.

### Cell Viability Assay

To examine the viability of LA-stimulated cells, we performed a colorimetric assay using a modification (30, 31) of the tetrazolium salt [MTT; (3-(4,5-dimethylthiazol-2-yl)-2,5-diphenyltetrazolium bromide)] method (32). Briefly, cells (THP-1 macrophages and iBMDM) in the medium dedicated for the cell line were plated (10^4^ cells/well; 100 µl) to triplicate wells of a 96-well flat-bottomed plate, and incubated in a humidified atmosphere at 37°C with 5% CO_2_ for 24 h. Cells were stimulated with 10, 1, 0.1 µg LA (1 mg/ml stock solution of LA in the medium supplemented with 5 µl of TEA) for 24 h. To determine viability, cells were incubated with 25 µl MTT (5 mg/ml stock solution) for 4 h at 37°C, and the medium was replaced with DMSO (100 µl). Absorbance was read at after 30 min at 570 nm. Cells incubated with medium were used as a reference samples.

### Stimulation of TNF-α, IL-1β, and IL-6 Release in Human Macrophages

Monocytic THP-1 cells were maintained in RPMI medium supplemented with 10% FCS (v/v) and differentiated into macrophages in medium supplemented with 500 nM/ml PMA (Merck) for 3 h at 37°C. The THP-1 macrophages were plated to 24-well tissue culture plates (Nunc) at a density of 0.5 × 10^6^ cells/well in 1 ml of RPMI medium and incubated for 7 days. The cells were then stimulated with LA (1, 0.1, 0.01, 0.001 µg) in 1 ml of medium. After 24 h, supernatants were collected and stored at −80°C for later determination of the concentrations of TNF-α, IL-1β, and IL-6. Unstimulated cultures supplemented with TEA were used as control samples. *E. coli* O55 LA was used as a positive control.

### Stimulation of TNF-α, IL-1β, and IL-6 Release in Murine Macrophages

Murine immortalized BMDM WT and iBMDM TLR4^−/−^ were plated to 24-well tissue culture plates (Nunc) at a density of 0.5 × 10^6^ cells/well in 1 ml of DMEM supplemented with 10% FCS and gentamycin (1:1000) (v/v), and incubated in a humidified atmosphere at 37°C with 5% CO_2_ for 24 h. Murine macrophages were stimulated with LA (1, 0.1, 0.01, 0.001 µg) in 1 ml of medium. Unstimulated cultures supplemented with TEA were used as control samples. *E. coli* O55 LA was used as a positive control. In all *in vitro* experiments, the same preparations of structurally well-defined ligands (LA O55 and LA I–IV) were used.

### Cytokines Analysis by ELISA

The concentrations of cytokines in supernatants collected from LA-stimulated human and murine macrophages were measured using human and murine TNF-α, IL-1β, and IL-6 ELISA kits according to the manufacturer’s instructions (BioLegend). In all *in vitro* experiments, the same preparations of structurally well-defined ligands (LA O55 and LA I–IV) were used.

### Data Analyses

Each experiment was repeated at least three times (from the stimulation stage) in duplicate. Data are presented as the median ± SD. The results were compared by one-way ANOVA with the Tukey–Kramer multiple comparison test, and differences were considered significant at *p* < 0.05 versus the control (TEA-supplemented cell cultures) or the appropriate LA type (in Figures 3 and 4).

## Results

### Isolation and Structural Analysis of LA

The screening of 85 *P. shigelloides* strains representing different O-serotypes was performed using MALDI-TOF MS; from the data, we identified the four strains (O14, O30, O61, O75) that produced the most homogeneous LA preparations (data not shown). Comparison of the obtained structural data with previously published data (8–10) enabled us to define three general types of *P. shigelloides* LA: LA I (serotype O14), LA II (serotype O30), LA III (serotype O61), and LA IV (O75). These *P. shigelloides* LA molecules were isolated by microextraction and analyzed by MALDI-TOF MS to validate sample purity.

**Figure 2:**
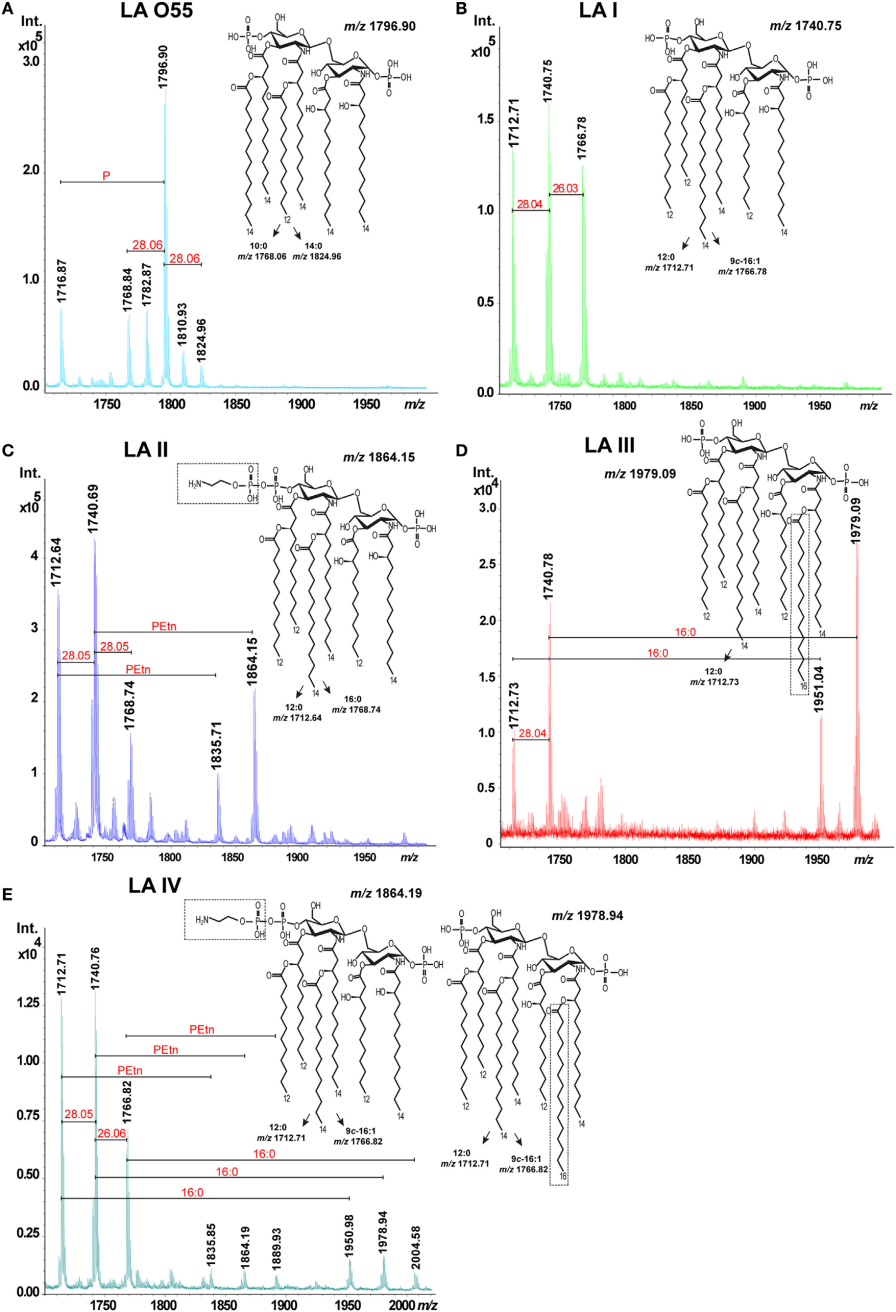
Matrix-assisted laser-desorption/ionization time-of-flight spectra of hexaacylated *Escherichia coli* O55 lipopolysaccharide (LA) **(A)** and *Plesiomonas shigelloides* LA I **(B)**, LA II **(C)**, LA III **(D)**, and LA IV **(E)**. Abbreviations: PEtn, phosphoethanolamine; and 16:0, hexadecanoic (palmitic) acid.

The mixed structures of LA I (Figure 2B, inset structure) were represented by ions detected at *m/z* 1712.71, *m/z* 1740.75, and *m/z* 1766.78. The ion at *m/z* 1740.75 was attributed to an asymmetric hexaacylated LA that is bisphosphorylated at O-1 and O-4’ and whose diglucosamine backbone is substituted by two amide-bound (*R*)-3-hydroxytetradecanoic acids (14:0[3-(*R*)-OH]) and four ester-bound fatty acids, as follows: two (*R*)-3-hydroxydodecanoic acids (12:0[3-(*R*)-OH]), one dodecanoic acid (12:0), and one tetradecanoic acid (14:0). The ions at *m/z* 1712.71 and *m/z* 1766.78 represented variants with different acyl lengths compared with the above-described structure: one with a mass difference of 28 Da, harboring a 12:0 instead of the 14:0; and one with a mass difference of 26 Da, harboring a 9*c*-16:1 instead of the 14:0 (10). No additional substitution, such as PEtn or 16:0, was identified for LA I.

The mixed structures of LA II (Figure 2C, inset structure) were represented by ions at *m/z* 1712.64, *m/z* 1740.69, *m/z* 1768.74, *m/z* 1835.71, and *m/z* 1864.15. The ions at *m/z* 1712.64 and *m/z* 1768.74 represented variants whose acyl lengths differed with those of the asymmetric hexaacylated and bisphosphorylated structure represented by the ion at *m/z* 1740.69 (see above). The ions at *m/z* 1835.71 and *m/z* 1864.15 represented variants that had PEtn substitutions (mass difference, ~123 Da) compared with the ions at *m/z* 1712.64 and *m/z* 1740.69, respectively. The total relative abundance of ions attributed to PEtn-substituted LA molecules was about 42%.

The mixed structures of LA III (Figure 2D, inset structure) were represented by ions at *m/z* 1712.73, *m/z* 1740.78 (representing the asymmetric hexaacylated and bisphosphorylated LA), *m/z* 1951.04, and *m/z* 1979.09 (the latter two representing the asymmetric heptaacylated and bisphosphorylated structures). The ion at *m/z* 1712.73 represented a variant that differed in its acyl length relative to that represented by the ion at *m/z* 1740.78 (as also seen in LA I and LA II). The ions at *m/z* 1951.04 and *m/z* 1979.09 represented heptaacylated variants that had 16:0 as a secondary acyl chain substitution (mass difference, ~238 Da) relative to those represented by the ions at *m/z* 1712.73 and *m/z* 1740.78, respectively. The total relative abundance of ions attributed to LA molecules with a 16:0 substitution was about 51%.

We also identified another LA variant type (*P. shigelloides* serotype O75, designated LA IV). The mixed structures of LA IV (Figure 2E, inset structures) were represented by ions at *m/z* 1712.71, *m/z* 1740.76, *m/z* 1766.82 (representing the asymmetric hexaacylated and bisphosphorylated structure with an altered acyl length as in LA I; total relative abundance among all ions, 89%), *m/z* 1835.85, *m/z* 1864.19, and *m/z* 1889.93 (representing variants of LA II differing in PEtn substitution; total relative abundance, 4%), *m/z* 1950.48, *m/z* 1978.94, and *m/z* 2004.58 (the latter three representing the asymmetric heptaacylated and bisphosphorylated structures of LA III that differed in their acyl lengths; total relative abundance, about 7%).

Lipopolysaccharide (LA) O55 was isolated by both microextraction and mild acid hydrolysis of commercially available LPS. Both LA O55 preparations revealed the same structure and heterogeneity. LA O55 (Figure 2A, inset structure) comprised a mixture of asymmetric hexaacylated and bisphosphorylated structures represented by ions at *m/z* 1768.84, *m/z* 1796.90, and *m/z* 1716.87 (attributed to the hexaacylated and phosphorylated structures). The main ion at *m/z* 1796.90 corresponded to a bisphosphorylated disaccharide backbone (β-D-Glc*p*N4P-(1→6)-α-D-Glc*p*N1P) substituted with the following primary and secondary fatty acids: 14:0[3-(*R*)-OH] at N-2 and O3; [14:0(3-(*R*)-*O*-12:0)] at N-2’; and [14:0(3-(*R*)-*O*-14:0)] at O-3’. The P-lacking counterpart of this structure was represented by the ion at *m/z* 1716.87. The ion at *m/z* 1768.84 represented a variant whose acyl length (mass difference, 28 Da, which corresponds to C_2_H_4_) differed in comparison with the variant represented by the ion at *m/z* 1796.90. No additional substitution, such as PEtn or 16:0, was identified for LA O55. The observed *E. coli* LA types were in agreement with the previously published data (5).

### Effect of the Various LA Types on TNF-α, IL-1β, and IL-6 Production in Murine Macrophages

**Figure 3:**
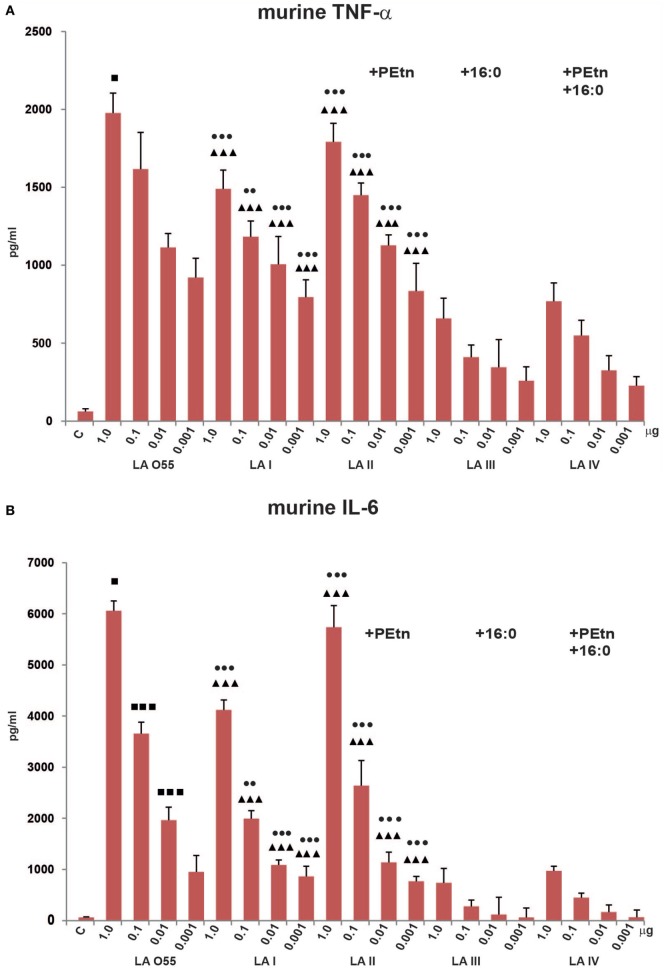
Stimulatory effect of *Plesiomonas shigelloides* LA (I-IV) on the productions of the proinflammatory cytokines, TNF-α **(A)**, and IL-6 **(B)**, by murine macrophages (BMDM WT). Statistically significant differences are marked with symbols: *p* < 0.05 (■/*/▲/●), *p* < 0.01 (■■/**/▲▲/●●), and *p* < 0.001 (■■■/***/▲▲▲/●●●), where ■, *, ▲, and ● refer to lipopolysaccharide (LA) I, LA II, LA III, and LA IV, respectively. *Escherichia coli* O55 LA (LA O55) was used as the positive control. C- Unstimulated cells cultured in the presence of triethalamine (TEA). *P* values were calculated by ANOVA.

To compare the effects of *in vitro* stimulation of murine iBMDM with *P. shigelloides* LA I-IV, we used ELISA to examine the releases of the proinflammatory cytokines, TNF-α, IL-1β, and IL-6 (Figure 3). Two preparations of *E. coli* O55 LA, one isolated by microextraction of the bacterial mass and one isolated by mild acid hydrolysis of commercially available O55 LPS, were used as positive controls and revealed similar stimulatory effects (data not shown for O55 LA isolated by mild acid hydrolysis of LPS). When TEA-supplemented medium was used as additional control, no biological effect was observed. Our MTT viability assay showed that the LA preparations (dose range, 1–0.01 µg of LA) had no toxic effect on cells. Experiments were performed using WT and TLR4^−/−^ murine macrophages. iBMDM TLR4^−/−^ did not demonstrate any measurable cytokine release upon stimulation with LA, which supports the involvement of the TLR4/MD-2 receptor complex in the LA-induced production of cytokines, and indicates that our preparations were free from other immunostimulants (Figure S1 in Supplementary Material).

We observed significant dose-dependent differences in the production levels of TNF-α and IL-6 by LA (I-IV)-stimulated murine macrophages compared with control (unstimulated) cells. No production of IL-1β was observed (data not shown), which is in agreement with the previous reports (33–35).

The bisphosphorylated, hexaacylated, and asymmetric forms of *E. coli* O55 and *P. shigelloides* LA I demonstrated similar abilities to induce murine macrophages to produce proinflammatory cytokines, although the production levels were slightly lower in cells treated with LA I. There was no significant difference in the amount of TNF-α produced by cells stimulated with LA O55 versus LA I for most of the tested LA concentrations, with the exception of the 1 µg dose (*p* < 0.05). However, LA I induced significantly less production of murine IL-6 than LA O55 at doses of 1.0, 0.1, and 0.01 µg (*p* < 0.05–0.001) (Figure 3). The observed trend can be explained only by the difference in the average length and saturation of the acyl groups. Moreover, a significant portion of the LA I preparation comprised the hexaacylated form with of 16:1 acyl chain instead of 14:0 or 12:0 (Figure 2B).

Phosphoethanolamine substitution (relative abundance 42%) did not influence the activity of LA II. No statistically significant differences were observed between LA I and LA II for both cytokines (Figure 3).

In comparison with bisphosphorylated and hexaacylated LA I, the presence of the seventh acyl chain of 16:0 (relative abundance 51%) in LA III had a profound effect on the *in vitro* productions of murine TNF-α and IL-6. LA III was at least approximately two- and approximately fivefold less active (*p* < 0.001) than hexaacylated LA I in triggering the productions of TNF-α (Figure 3A) and IL-6 (Figure 3B), respectively.

The presence of PEtn (4%) and the decreased contribution of the 16:0 acyl chain (7%) were sufficient to attenuate the activity LA IV in a manner similar to that seen for LA III; there was no significant difference in the abilities of LA III and LA IV to trigger the production of TNF-α and IL-6 (Figure 3).

### Effect of the Various LA Types on TNF-α, IL-1β, and IL-6 Production in Human Macrophages

**Figure 4:**
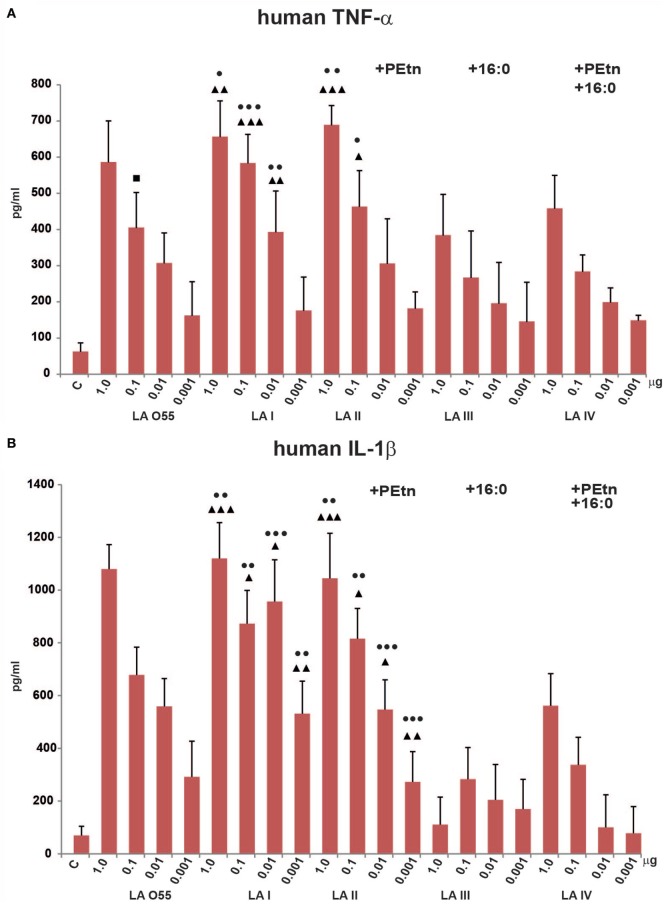

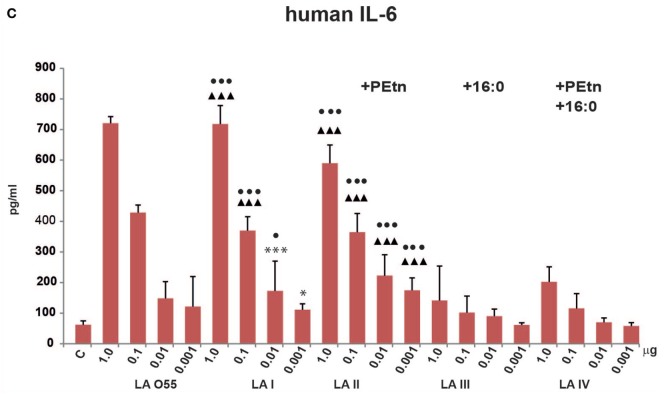
Stimulatory effect of *Plesiomonas shigelloides* LA (I-IV) on the productions of the proinflammatory cytokines, TNF-α **(A)**, IL-1β **(B)**, and IL-6 **(C)**, by differentiated human THP-1 macrophages. Statistically significant differences are marked with symbols: *p* < 0.05 (■/*/▲/●), *p* < 0.01 (■■/**/▲▲/●●), and *p* < 0.001 (■■■/***/▲▲▲/●●●), where ■, *, ▲, and ● refer to lipopolysaccharide (LA) I, LA II, LA III, and LA IV, respectively. *Escherichia coli* O55 LA (LA O55) was used as the positive control. C- Unstimulated cells cultured in the presence of triethalamine (TEA). *P* values were calculated by ANOVA.

We next compared the abilities of *P. shigelloides* LA (I–IV) to stimulate the productions of TNF-α, IL-1β, and IL-6 by humanTHP-1 macrophages *in vitro* (Figure 4). Our MTT viability assay showed that the LA preparations (dose range, 1–0.01 µg of LA) had no toxic effect on cells. The tested ligands (LA I-IV, LA O55) dose-dependently stimulated human macrophages in most cases, although some deviations were seen for production of IL-1β by cells treated with 0.01 µg LA I or 1.0 µg LA III (Figure 4B).

*Escherichia coli* O55 LA and *P. shigelloides* LA I (Figure 2A,B) demonstrated similar abilities to induce proinflammatory cytokine production by human macrophages. No significant difference was observed in the abilities of LA O55 and LA I to induce the productions of TNF-α, IL-1β, and IL-6 at any tested dose (Figure 4), with the exception of the TNF-α production triggered by 0.1 µg LA (*p* < 0.05) (Figure 4A).

Phosphoethanolamine substitution (relative abundance 42%) did not influence the ability of LA II to stimulate the productions of TNF-α or IL-1β (Figures 4A,B). For IL-6, doses of 0.01 and 0.001 µg yielded higher cytokine production for cells treated with LA II versus LA I (*p* < 0.05–0.001) (Figure 4C).

The palmitoylation that was characteristic of LA III (relative abundance 51%) significantly decreased the *in vitro* effects (*p* < 0.05–0.001) of most of LA doses on the productions of TNF-α, IL-1β, and IL-6 in comparison to those triggered by the same doses of LA I (Figure 4), with the exception of 0.001 µg for TNF-α (Figure 4A) and 0.01 and 0.001 µg for IL-6 (Figure 4C).

The presence of PEtn (4%) and the decreased contribution of the 16:0 component (7%) were sufficient to attenuate the activity of LA IV in a manner similar to that seen for LA III; there was no significant difference in the cytokine productions triggered by the tested doses of LA III and LA IV (Figure 4).

## Discussion

Recognition of LPS, a major component of the outer membrane of Gram-negative bacteria, by the TLR4/MD-2 complex is essential for the host’s ability to control bacterial infection. The binding of an LA molecule by the co-receptor protein MD-2, initiates a proinflammatory signaling cascade to trigger the innate immune response of the host. Unbalanced immune response of LPS-responsive cells may result in sepsis syndrome. Studies on structure–activity relationships can help clarify how structural modifications of LPS/LA contribute to bacterial pathogeneses. Indeed, understanding the structural requirements for the biological activity of LA has led to the development of effective anti-inflammatory agents (4, 23, 36).

Natural *P. shigelloides* LA molecules comprise a mixture of structures that differ in the numbers, lengths, and saturations of their acyl chains, as well as in PEtn substitutions. Following our screening of 85 O-serotypes of *P. shigelloides*, we obtained native LA preparations (LA I–IV) from four strains of different O-serotypes. In comparison with the bisphosphorylated, hexaacylated and asymmetric *E. coli* O55 LA, the preparations differed in their fatty acid lengths (LA I), PEtn substitutions (LA II), and 16:0 substitutions (LA III), with LA IV showing a particularly low contribution of PEtn and 16:0 within its structure. AraN or uronic acid substitutions and very long fatty acids were not identified for *P. shigelloides* LA.

Comparing the activity of the bisphosphorylated, hexaacylated, and asymmetric LA I with that of *E. coli* O55 LA demonstrated that decreasing the chain length to two to four acyl groups of 12 carbon atoms or two acyl groups of 12 carbon atoms and the presence of the one 16:1 (9*c*-16:1) (Figures 2A,B) (19) did not influence the *in vitro* activity of LA I in murine or human macrophages. LA I seemed to be a slightly weaker activator of murine macrophages, although not to a statistically significant degree. As mentioned above, the general structural differences between LA O55 and LA I (both of which are bisphosphorylated and hexaacylated) include a difference in acylation, with the latter having a higher contribution of shorter [12:0/12:(3-OH) instead of 14:0/14:(3-OH)] and unsaturated (9*c*-16:1 instead of 14:0 or 12:0) acyl residues on an identical bisphosphorylated disaccharide backbone. Little published data have focused on the structure–activity relationships of LA with direct reference to the acyl chain lengths. Generally, shorter acyl chains improve LA solubility and decrease aggregate formation improving cell activation. However, too long or too short acyl chains could impair effective LA/TLR4/MD-2 interactions (27). A previous comparison of TNF-α production in murine BMDM stimulated with LPS from *E. coli, Y. pestis*, and *Psychrobacter cryohalolentis* showed that shortening the average acyl chain length to 10–12 carbon atoms (as seen for *P. cryohalolentis*) slightly attenuated the *in vitro* activity of whole LPS. Moreover similar TNF-α productions were stimulated by the LPS of *E. coli* and that of *Y. pestis*, which has bisphosphorylated hexaacylated LA molecules substituted with two l-Ara4N residues and 9*c*-16:1 instead of 12:0 (37). Consistent with this, we herein found that the presence of 9*c*-16:1 in *P. shigelloides* LA I did not influence the tested biological activity in human and murine macrophages. By contrast, Zhang et al. investigated a synthetic analog of bisphosphorylated, hexaacylated, and asymmetric *E. coli* LA (5 acyl chains of 14 carbon atoms and 1 of 12 carbon atoms) and a counterpart built of shorter acyl chains (2 acyl chains of 14 carbon atoms and 4 of 12 carbon atoms), and found that the latter showed higher potency in stimulating the productions of TNF-α, IL-1β, and IL-6 by murine macrophages (38). This discrepancy among the published data for natural LA preparations may be explained by small differences in the length between acyl chains built of 10/12 and 14 carbon atoms that depending on host cell and co-existing forms of LA with additional substituents in native preparations may decrease or increase *in vitro* activity of LA. It was suggested that fatty acid chain length within 12–14 carbon atoms ensures biological activity maintenance. Despite the small differences in the average acyl chain lengths, all of the discussed examples have the bisphosphorylated, hexaacylated, and asymmetric structure that is the most important prerequisite for the interaction between LA and cell receptor. Conversely, little or no endotoxic activity was found for LA carrying more extended acyl residues (at least 18-carbon long) and branched and unsaturated acyl residues (e.g., *Legionella pneumophila* LA with the very-long-chain fatty acid, 27-hydroxyoctacosanoic acid) (1).

The PEtn decoration of *P. shigelloides* LA II did not impact its biological activity on murine and human macrophages. In general, no significant difference was observed in the activities of LA I and LA II, except that the production of IL-6 by human macrophages was significantly increased by 0.01 or 0.001 µg of LA II. The two phosphate groups of LA are known to be critical for complete activation of the macrophages, and an additional PEtn substitution was shown to affect the biological activity of *Cronobacter sakazakii* LPS (39, 40). The presence of PEtn and l-Ara4N moieties in LA decreases its net negative charge, which has been shown to increase resistance to polymixin B and other cationic antimicrobial peptides (CAMPs), as well as to complement-mediated killing (10, 25, 26). Thus, for classical LA structures (*E. coli*-like), PEtn substitution seems to be an important factor for other activities than TNF-α, IL-1β, and IL-6 production by macrophages. The degree of LA phosphorylation and phosphoethanolaminylation can be correlated to inflammatory potential of bacteria and ability to induce immune tolerance *in vitro* (41). For example, *N. meningitidis* LA mixture of hexaacylated forms with 2P and PEtn, 3P, or 2P, along with apparently less abundant pentaacyl LA molecules with 2P, was more active to trigger proinflammatory cytokine response of THP-1 cells than the hexaacylated and bisphosphorylated LA. This suggested that increasing the number of phosphoryl substituents increases the potency of TNF-α induction. Moreover, the decreases in P and PEtn substitution observed for invasive isolates of *N. meningitidis* were correlated with increased occurrence of septicemia. The most highly phosphorylated bacterial isolates had relatively diminished ability to survive systemically and cause systemic disease (42). Among the tested structures, only the bisphosphorylated and heptaacylated LA III structure decorated with 16:0 showed a significant alteration in the proinflammatory activity. LA III triggered significantly less production of all tested cytokines in both murine and human macrophages: it was at least ~2–5- and 2–4-fold less active than hexaacylated LAI and O55 LA in murine and human macrophages, respectively. In the LA molecules of *E. coli* and *P. shigelloides*, palmitoylation is related to the addition of a palmitate (16:0) to the 14:0(3-OH) at N-2 of the α-D-Glc*p*N residue within the carbohydrate backbone structure (12, 29, 43–45).

Palmitoylation and the addition of PEtn are dependent on environmental conditions and can directly protect the bacterium against host immune defenses. Such modification alters the ability of LA to activate defense mechanisms through TLR4/MD-2 receptor complex-mediated signal transduction (4, 46–48). Heptaacylated native LA of *S. enterica* sv. Minnesota Re deep rough mutant strain (R595) and the synthetic analog, compound 516, were shown to moderately induce the inductions of TNF-α, IL-1β, and IL-6 in human monocytes (THP-1 cell line) (48). In some studies, compound 516-treated human mononuclear cells from healthy donors or isolated monocytes/macrophages exhibited 10- to 100-fold decreases in IL-1 production compared to those triggered by *E. coli* O55 and its synthetic analog, compound 506 (8, 49). For murine macrophages, the ability of LA to trigger TNF-α and IL-1β production was also significantly decreased for synthetic analogs of the bisphosphorylated and asymmetric heptaacylated LA of *S. enterica* sv. Typhimurium compared to synthetic bisphosphorylated and asymmetric hexaacylated *E. coli* LA (38). Palmitoylation has been well described for *S. enterica* sv. Typhimurium, where the PhoP/PhoQ regulatory system palmitoylates LA in response to environmental conditions (50). In response to specific environmental signals, PhoQ phosphorylates PhoP, leading to the activation or repression of over 40 different genes, including *pagP*, which encodes a palmitoyltransferase responsible for transferring palmitate to LA. The same study demonstrated that the addition of palmitate provides resistance to certain CAMPs by increasing the integrity of the outer membrane and preventing the translocation of CAMPs across the bilayer (46, 50). Palmitoylation has also been described in *E. coli* and *K. pneumoniae*, and similar modifications at various positions in the LA structure have been described for *Legionella pneumophila, Pseudomonas aeruginosa, Bordetella bronchiseptica, Y. enterocolitica*, and *Y. pseudotuberculosis* LPS (19).

This is the first report concerning the biological activity of *P. shigelloides* LA with respect to structure–activity relationships. Even though they have relatively short acyl chains and unsaturated acyl residue (16:1), the bisphosphorylated, hexaacylated, and asymmetric forms of *P. shigelloides* LA represent a highly immunostimulatory structure. Moreover, similar trends in *in vitro* activity of LA I-IV were observed for human and murine macrophages, what was generally in agreement with previously published data. An interaction between LA/LPS and TLR4/MD-2 complex is species-specific, where depending on species (e.g., human or murine MD-2) the same LA modification may elicit opposed host response. Moreover, several key differences in the amino acid sequences of human and murine TLR4/MD-2 receptor complexes have been shown to alter their ability to recognize different types of LA (27). According to the literature, most important structural features of LA for recognition by the TLR4 complex are acyl chains number and pattern (symmetric vs. asymmetric), and the presence of very short or long acyl chains (27). Five of the acyl chains are buried in a hydrophobic cavity of the human TLR4 adaptor molecule, MD-2, while the sixth chain mediates TLR4 binding and heterodimerization, and subsequent intracellular signaling (27, 51, 52). In general, different behavior of human and murine cells was observed for tetraacylated forms. We have shown that in laboratory conditions *P. shigelloides* does not synthesize penta- or tetraacylated forms. By searching for the diversity of *P. shigelloides* LA, we have shown that this species is able to utilize some LA modification systems that might be useful to evade the immune response, for example, palmitoylation and PEtn substitution. Even though PEtn substitution does not influence *in vitro* cytokine induction of isolated LA, it may increase resistance of the bacteria against CAMPs. Heptaacylated forms may promote bacterial survival by decreasing cytokine production. Thus, natural *P. shigelloides* LA heterogeneity may be prominent for the immune system modulation and pathogenesis, since this species was shown to be able to adhere, internalize and multiply within mammalian cells *in vitro* (16, 53).

## Author Contributions

MK, JS, and CL analyzed the literature and designed the research. MK and MW performed experiments. MK, JL, and CL wrote the manuscript. All authors analyzed the data.

## Conflict of Interest Statement

The authors declare that the research was conducted in the absence of any commercial or financial relationships that could be construed as a potential conflict of interest.

## Supplementary Material

Figure S1 Stimulatory effect of *P. shigelloides* LA (I-IV) on the production of the proinflammatory cytokine TNF-α **(A)** and IL-6 **(B)** by TLR4^−/−^ murine macrophages (iBMDM).

## References

[1] Alexander C Zähringer U Chemical structure of lipid A – the primary immunomodulatory center of bacterial lipopolysaccharides. Trends Glycosci Glyc (2002) 14:69–86. 10.4052/tigg.14.69

[2] Takeuchi O Akira S Pattern recognition receptors and inflammation. Cell (2010) 140:805–20. 10.1016/j.cell.2010.01.022 20303872

[3] Meashima N Fernandez RC Recognition of lipid A by the TLR4-MD-2 receptor complex. Front Cell Infect Microbiol (2013) 3:3 10.3389/fcimb.2013.00003 23408095 PMC3569842

[4] Artner D Oblak A Ittig S Garate JA Horvat S Arrieumerlou C Conformationally constrained lipid A mimetics for exploration of structural basis of TLR4/MD-2 activation by lipopolysaccharide. Chem Biol (2013) 8:2423–32. 10.1021/cb4003199 PMC3833292 23952219

[5] Raetz CRH Purcell S Mayer MV Qureshi N Takayama K. Isolation and characterization of eight lipid A precursors from a 3-deoxy-D-*manno*-octylosonic acid-deficient mutant of *Salmonella typhimurium*. J Biol Chem (1985) 260:16080–8. 3905804

[6] Raetz CR Reynolds CM Trent MS Bishop RE. Lipid A modification systems in gram-negative bacteria. Annu Rev Biochem (2007) 76:295–329. 10.1146/annurev.biochem.76.010307.145803 17362200 PMC2569861

[7] Imoto M Shiba T Naoki H Iwashita T Rietschel ET Wollenweber HW Chemical structure of *E. coli* lipid A: linkage site of acyl groups in the disaccharide backbone. Tetrahedron Lett (1983) 24:4017–20. 10.1016/S0040-4039(00)88251-9

[8] Loppnow HL Brade L Brade H Rietschel ET Kusumoto S Shiba T Induction of human interleukin 1 by bacterial and synthetic lipid A. Eur J Immunol (1986) 16:126301267. 10.1002/eji.1830161013 3490388

[9] SenGupta S Hittle LE Ernst RK Uriarte SM Mitchell TC. A *Pseudomonas aeruginosa* hepta-acylated lipid A variant associated with cystic fibrosis selectively activates human neutrophils. J Leukoc Biol (2016) 100(5):1047–59. 10.1189/jlb.4VMA0316-101R 27538572 PMC6608067

[10] Lewis LA Choudhury B Balthazar JT Martin LE Ram S Rice PA Phoshoethanolamine substitution of lipid A and resistance of *Neisseria gonorrhoeae* to cationic antimicrobial peptides and complement-mediated killing by normal human serum. Infect Immun (2009) 77:1112–20. 10.1128/IAI.01280-08 19114544 PMC2643632

[11] Steimle A Autenrieth IB Frick JS. Structure and function: lipid A modifications in commensals and pathogens. Int J Med Microbiol (2016) 306(5):290–301. 10.1016/j.ijmm.2016.03.001 27009633

[12] Caroff M Karibian D Cavaillon JM Haeffner-Cavaillon N. Structural and functional analyses of bacterial lipopolysaccharides. Microbes Infect (2002) 4(9):915–26. 10.1016/S1286-4579(02)01612-X 12106784

[13] Chen X Chen Y Young Q Kong H Yu F Han D *Plesiomonas shigelloides* infection in southeast China. PLoS One (2013) 8(11):e77877. 10.1371/journal.pone.0077877 24223738 PMC3817182

[14] Auxiliadora-Martins M Bellissimo-Rodrigues F Viana JM Teixiera GCA Nicolini EA Cordeiro KSM Septic shock caused by *Plesiomonas shigelloides* in a patient with sickle beta-zero thalassemia. Heart Lung (2010) 39(4):335–9. 10.1016/j.hrtlng.2009.06.015 20561842

[15] Stock I *Plesiomonas shigelloides*: an emerging pathogen with unusual properties. Rev Med Microbiol (2004) 15:129–39. 10.1097/00013542-200410000-00002

[16] Janda JM Abbott SL McIver CJ. *Plesiomonas shigelloides* revisited. Clin Microbiol Rev (2016) 29(2):349–74. 10.1128/CMR.00103-15 26960939 PMC4786884

[17] Lukasiewicz J Niedziela T Jachymek W Kenne L Lugowski C Structure of the lipid A-inner core region and biological activity od *Plesimonas shigelloides* O54 (strain CNCTC 113/92) lipopolysaccharide. Glycobiology (2006) 16(6):538–50. 10.1093/glycob/cwj094 16490765

[18] Kaszowska M Jachymek W Lukasiewicz J Niedziela T Kenne L Lugowski C. The unique structure of complete lipopolysaccharide isolated from semi-rough *Plesiomonas shigelloides* O37 (strain CNCTC 39/89) containing (2*S*)-*O*-(4-oxopentanoic acid)-α-D-Glc*p* (α-D-Lenose). Carbohydr Res (2013) 378:98–107. 10.1016/j.carres.2013.04.015 23711248

[19] Lukasiewicz J Dzieciatkowska M Niedziela T Jachymek W Augustyniuk A Kenne L Complete lipopolysaccharide of *Plesiomonas shigelloides* O74:H5 (strain CNCTC 144/92). Lipid A, its structural variability, the linkage to the core oligosaccharide, and the biological activity of the lipopolysaccharide. Biochemistry (2006) 45:10434–47. 10.1021/bi060774d 16939196

[20] Schromm AB Brandenburg K Loppnow H Moran AP Koch MH Rietschel ET Biological activities of lipopolysaccharides are determined by the shape of their lipid A portion. Eur J Biochem (2000) 267:2008–13. 10.1046/j.1432-1327.2000.01204.x 10727940

[21] Kusumoto S Fukase K Fukase Y Kataoka M Yoshizaki H Sato K Structural basis for endotoxic and antagonistic activities: investigation with novel synthetic lipid A analogs. J Endotoxin Res (2003) 9(6):361–6. 10.1177/09680519030090060901 14733722

[22] Muroi M Tanamoto K. Structural regions of MD-2 that determine the agonist-antagonist activity of lipid IVa. J Biol Chem (2006) 281(9):5484–591. 10.1074/jbc.M509193200 16407172

[23] Fujimoto Y Adachi Y Akamatsu M Fukase Y Kataoka M Suda Y Synthesis of lipid A and its analogues for investigation of the structural basis for their bioactivity. J Endotoxin Res (2005) 11(6):341–7. 10.1179/096805105X76841 16303089

[24] Akashi S Nagai Y Ogata H Oikawa M Fukase K Kusumoto S Human MD-2 confers on mouse toll-like receptor 4 species-specific lipopolysaccharide recognition. Int Immunol (2001) 13(12):1595–9. 10.1093/intimm/13.12.1595 11717200

[25] Packiam M Yedery RD Begum AA Carlson RW Ganguly J Sempowski GD Phosphoethanolamine decoration of *Neisseria gonorrhoeae* lipid A plays a dual immunostimulatory and protective role during experimental genital tract infection. Infect Immun (2014) 82(6):2170–9. 10.1128/IAI.01504-14 24686069 PMC4019182

[26] Zughaier SM Kandler JL Balthazar JT Shafer WM. Phosphoethanolamine modification of *Neisseria gonorrhoeae* lipid A reduces autophagy flux in macrophages. PLoS One (2015) 10(12):e0144347. 10.1371/journal.pone.0144347 26641098 PMC4671640

[27] Oblak A Jerala R. The molecular mechanism of species-specific recognition of lipopolysaccharides by the MD-2/TLR4 receptor complex. Mol Immunol (2015) 63:134–42. 10.1016/j.molimm.2014.06.034 25037631

[28] El Hamidi A Tirsoaga A Novikov A Hussein A Caroff M. Microextraction of bacterial lipid A: easy and rapid method for mass spectrometric characterization. J Lipid Res (2005) 46:1773–8. 10.1194/jlr.D500014-JLR200 15930524

[29] Lukasiewicz J Jachymek W Niedziela T Kenne L Lugowski C. Structural analysis of the lipid A isolated from *Hafnia alvei* 32 and PCM 1192 lipopolysaccharides. J Lipid Res (2010) 51:564–74. 10.1194/jlr.M001362 19706748 PMC2817586

[30] Hansen MB Nielsen SE Berg K. Re-examination and further development of a precise and rapid dye method for measuring cell growth/cell kill. J Immunol Methods (1989) 119:203–10. 10.1016/0022-1759(89)90397-9 2470825

[31] Vistica DT Skehan P Scudiero D Monks A Pittman A Boyd MR. Tetrazolium-based assays for cellular viability: a critical examination of selected parameters affecting formazan production. Cancer Res (1991) 51(10):2515–20. 2021931

[32] Mosmann T. Rapid colorimetric assay for cellular growth and survival: application to proliferation and cytotoxicity assays. J Immunol Methods (1983) 65:55–63. 10.1016/0022-1759(83)90303-4 6606682

[33] He Y Franchi L Núñez G. TLR agonists stimulate Nlrp3-dependent IL-1β production independently of the purinergic P2X7 receptor in dendritic cells and *in vivo*. J Immunol (2013) 190(1):334–9. 10.4049/jimmunol.1202737 23225887 PMC3531812

[34] Karmakar M Katsnelson MA Dubyak GR Pearlman E. Neutrophil P2X7 receptors mediate NLRP3 inflammasome-dependent IL-1β secretion in response to ATP. Nat Commun (2016) 7:10555. 10.1038/ncomms10555 26877061 PMC4756306

[35] Netea MG Nold-Petry CA Nold MF Joosten LA Opitz B van der Me JH Differential requirement for the activation of the inflammasome for processing and release of IL-1beta in monocytes and macrophages. Blood (2009) 113(10):2324–35. 10.1182/blood-2008-03-146720 19104081 PMC2652374

[36] Molinaro A Holst O Di Lorenzo F Callaghan M Nurisso A D’Errico G Chemistry of lipid A: at the heart of innate immunity. Chemistry (2015) 21(2):500–19. 10.1002/chem.201403923 25353096

[37] Korneev KV Kondakova AN Arbatsky NP Novototskaya-Vlasova KA Rivkina EM Anisimov AP Distinct biological activity of lipopolysaccharides with different lipid A acylation status from mutant strains of *Yersinia pestis* and some members of genus *Psychrobacter*. Biochemistry (Mosc) (2014) 79(12):1333–8. 10.1134/S0006297914120062 25716726

[38] Zhang Y Gaekwad J Wolfert MA Boons GJ. Modulation of innate immune responses with synthetic lipid A derivatives. J Am Chem Soc (2007) 129(16):5200–16. 10.1021/ja068922a 17391035 PMC2529018

[39] Liu L Li Y Wang X Guo W A phosphoethanolamine transferase specific for the 4’-phosphate residue of *Cronobacter sakazakii* lipid A. J Appl Microbiol (2016) 121:1444–56. 10.1111/jam.13280 27564119

[40] Renzi F Zähringer U Chandler CE Ernst RK Cornelis GR Ittig SJ. Modification of the 1-phosphate group during biosynthesis of *Capnocytophaga canimorsus* lipid A. Infect Immun (2015) 84(2):550–61. 10.1128/IAI.01006-15 26644381 PMC4730577

[41] John CM Phillips NJ Din R Liu M Rosenqvist E Høiby EA Lipooligosaccharide structures of invasive and carrier isolates of *Neisseria meningitidis* are correlated with pathogenicity and carriage. J Biol Chem (2016) 291(7):3224–38. 10.1074/jbc.M115.666214 26655715 PMC4751370

[42] John CM Liu M Jarvis GA. Natural phosphoryl and acyl variants of lipid A from *Neisseria meningitidis* strain 89I differentially induce tumor necrosis factor-alpha in human monocytes. J Biol Chem (2009) 284(32):21515–25. 10.1074/jbc.M109.004887 19531474 PMC2755876

[43] Guo LKB Lim CM Poduje M Daniel JS Gunn M Hackett M Lipid A acylation and bacterial resistance against vertebrate antimicrobial peptides. Cell (1998) 95:189–98. 10.1016/S0092-8674(00)81750-X 9790526

[44] Lamarche MG Kim SH Crepin S Mourez M Bertrand N Bishop RE Modulation of hexa-acyl pyrophosphate lipid A population under *Escherichia coli* phosphate (Pho) regulon activation. J Bacteriol (2008) 190:5256–64. 10.1128/JB.01536-07 18515419 PMC2493271

[45] Helander IM Kato Y Kilpelainem I Kostiainem R Lindner B Nummila K Characterization of lipopolysaccharides of polymyxin-resistant and polymyxin-sensitive *Klebsiella pneumonia*e O3. Eur J Biochem (1996) 237:272–8. 10.1111/j.1432-1033.1996.0272n.x 8620884

[46] Bishop RE The lipid A palmitoylotransferase PagP: molecular mechanisms and role in bacterial pathogenesis. Mol Microbiol (2005) 57:900–12. 10.1111/j.1365-2958.2005.04711.x 16091033

[47] Bishop RE Kim SH El Zoeiby A. Role of lipid A palmitoylation in bacterial pathogenesis. J Endotoxin Res (2005) 11(3):174–80. 10.1177/09680519050110030601 15949146

[48] Janusch H Brecker L Lindner B Alexander C Gronow S Heine H Structural and biological characterization of highly purified hepta-acyl lipid a present in the lipopolysaccharide of the *Salmonella enterica* sv. minnesota Re deep rough mutant strain R595. J Endotoxin Res (2002) 8(5):343–56. 10.1177/09680519020080050801 12537693

[49] Feist W Ulmer AJ Musehold J Brade H Kusumoto S Flad HD. Induction of tumor necrosis factor-alpha release by lipopolysaccharide and defined lipopolysaccharide partial structures. Immunobiology (1989) 179(4–5):293–307. 10.1016/S0171-2985(89)80036-1 2613271

[50] Kawasaki K Ernst RK Miller SI. 3-O-deacylation of lipid A by PagL, a PhoP/PhoQ-regulated deacylase of *Salmonella typhimurium*, modulates signaling through toll-like receptor 4. J Biol Chem (2004) 279(19):20044–8. 10.1074/jbc.M401275200 15014080

[51] Maeshima N Fernandez RC. Recognition of lipid A variants by the TLR4-MD-2 receptor complex. Front Cell Infect Microbiol (2013) 3:1–13. 10.3389/fcimb.2013.00003 23408095 PMC3569842

[52] Park BS Song DH Kim HM Choi BS Lee H Lee JO The structural basis of lipopolysaccharide recognition by the TLR4/MD-2 complex. Nature (2009) 458:1191–5. 10.1038/nature07830 19252480

[53] Theodoropoulos C Wong TH O’Brien M Stenzel D. *Plesiomonas shigelloides* enters polarized human intestinal Caco-2 cells in an *in vitro* model system. Infect Immun (2001) 69(4):2260–9. 10.1128/IAI.69.4.2260-2269.2001 11254582 PMC98154

