# Supplementary figures and images for: Structure–Activity Relationship of Plesiomonas shigelloides Lipid A to the Production of TNF-α, IL-1β, and IL-6 by Human and Murine Macrophages
Source: Front Immunol. 2017 Dec 11;8:1741. doi: 10.3389/fimmu.2017.01741 (PMC5732152; doi:10.3389/fimmu.2017.01741)

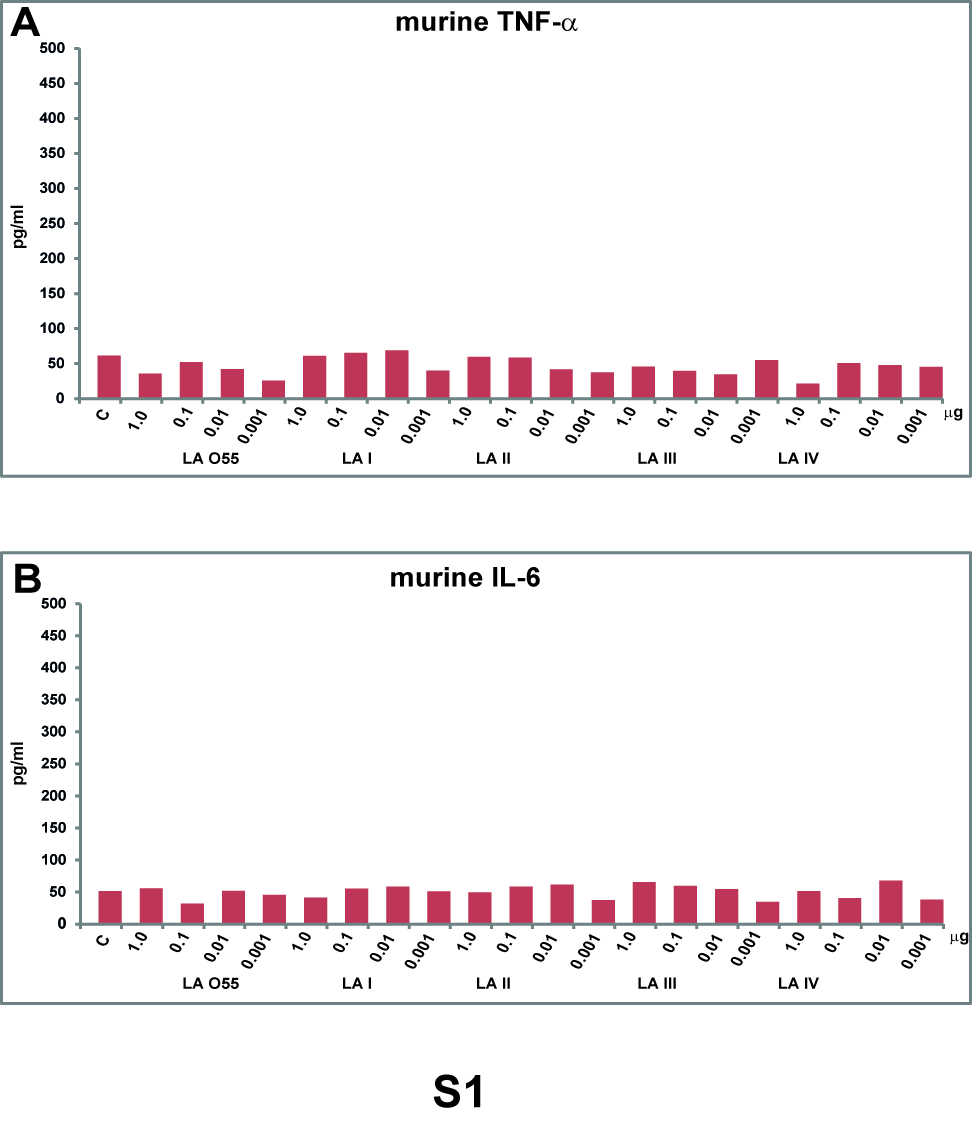

Supplement: Figure S1 — Stimulatory effect of P. shigelloides LA (I-IV) on the production of the proinflammatory cytokine TNF-α (A) and IL-6 (B) by TLR4−/− murine macrophages (iBMDM). [file Image_1.tif]
